# ﻿Re-collected after 55 years: a new species of *Bembidion* (Coleoptera, Carabidae) from California

**DOI:** 10.3897/zookeys.1156.101072

**Published:** 2023-03-27

**Authors:** David R. Maddison, John S. Sproul, Kipling Will

**Affiliations:** 1 Department of Integrative Biology, Oregon State University, Corvallis, OR 97331, USA Oregon State University Corvallis United States of America; 2 Department of Biology, University of Nebraska Omaha, Omaha, NE 68182, USA University of Nebraska Omaha Omaha United States of America; 3 Essig Museum of Entomology, University of California, Berkeley, CA 94720, USA University of California Berkeley United States of America

**Keywords:** Bembidiini, DNA, ground beetle, molecular systematics, morphology, phylogeny, Trechinae

## Abstract

A new species of the carabid beetle genus *Bembidion* Latreille is described from the Central Valley, Los Angeles Basin, and surrounding areas of California. *Bembidionbrownorum***sp. nov.** is a distinctive species, a relatively large member of the subgenus Notaphus Dejean, and within *Notaphus* a member of the *B.obtusangulum* LeConte species group. It has faint spots on the elytra and a large, convex, rounded prothorax. Of the 22 specimens from 11 localities, all but one were collected more than 55 years ago. Although the collection of the holotype in 2021 at UV light suggest the species is still extant, the lack of other recent specimens suggests the species may have a more restricted distribution than in the past, and its populations may be in decline.

## ﻿Introduction

Our knowledge of the North American carabid (ground beetle) fauna has benefitted from many decades of significant study and publication (e.g., Hatch 1953; Lindroth 1961–1969; Ball and Bousquet 2000; Bousquet 2012). However, the carabid fauna of the western and southern regions of the continent remains understudied, with distributional ranges, habitats, and life histories of the carabids in those regions still poorly documented and new species still to be discovered and described. To fill this knowledge gap, we have been sampling carabids in the western United States and Canada, frequently targeting areas that appear to have been little sampled.

With about 275 species described from the USA and Canada, *Bembidion* Latreille is the largest genus of carabid beetles in the region (and the world), and one of the groups most likely to contain undiscovered taxa (e.g., Maddison and Cooper 2014; Sproul and Maddison 2017a; Maddison 2020). These are small beetles, with adults of most species ranging between 3 and 6 mm in length. The majority of species live along the edges of bodies of water, from ocean shores and estuarian areas to pond shores and marshes, river and creek shores, and high-elevation snow fields, but some species are associated with grasslands, alpine meadows, and other areas far from open water. California has the richest fauna in North America, with over 120 species known.

Although within an hour’s drive of the major metropolitan center of Sacramento, many parts of Colusa County, California remain unsampled and little represented in major California entomology collections. Most of the land in the county is privately owned and used for agricultural production, which limits opportunities for access and sampling. Somewhat serendipitously, access and permission to collect was obtained for a ranch, known locally as “Mountain House,” in Colusa County. Over the course of two years, periodic sampling was conducted to determine the diversity of carabid beetles on the property.

**Figure 1.**
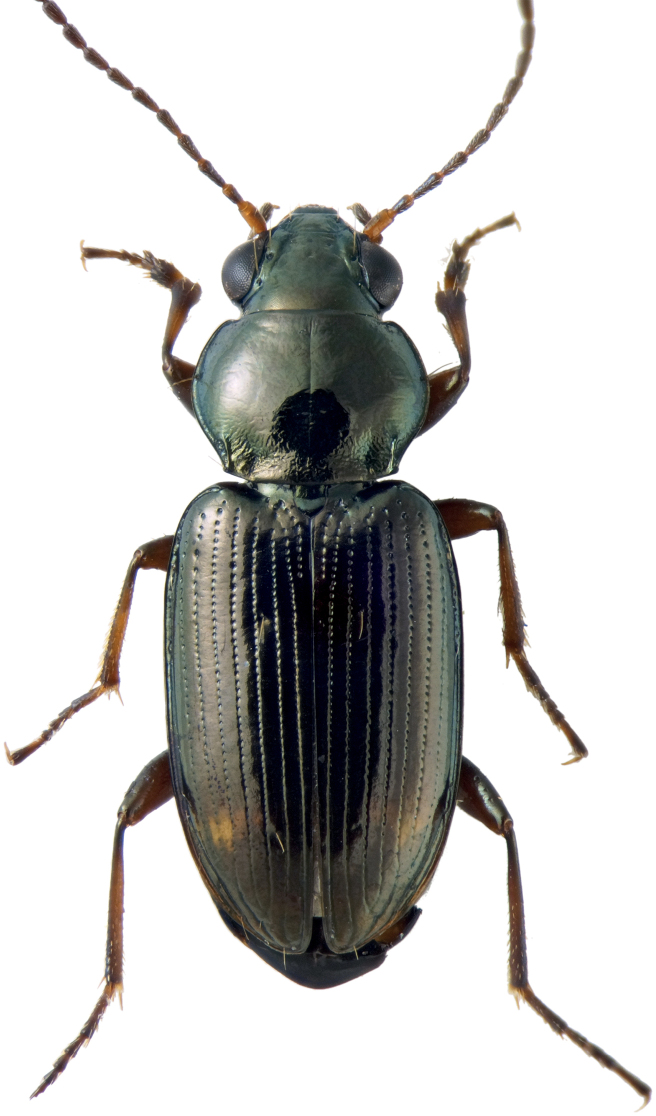
Holotype male of *Bembidionbrownorum*.

Among the many insects sampled, a single specimen of *Bembidion* stood out as very distinctive (Fig. 1). It was somewhat similar in overall appearance to *Bembidionmormon* Hayward, *B.obtusangulum* LeConte, and *B.callens* Casey, all members of the subgenus Notaphus Dejean, but the specimen’s morphological characteristics were unusual enough that it seemed likely that it belonged to a previously unknown species. Results of analyses of DNA sequence data from the specimen provided additional evidence that it was a new species.

The discovery of this single specimen motivated a search in major carabid collections in California for additional specimens. Only 21 additional specimens were located, all of which were collected more than 55 years ago. The distribution of specimens suggests this was a widespread species, but the lack of recently collected specimens suggests it may now be more restricted in distribution. Given the prospect of a declining and potentially threatened species, we felt it urgent to describe this species to spur the search for additional populations, and prompt research to better understand the species.

## ﻿Materials and methods

Members of *Bembidion* were examined from the collections listed below. Each collection’s listing begins with the code used in the text.

**BMEC** Bohart Museum of Entomology, University of California, Davis, USA;

**CAS**California Academy of Sciences, San Francisco, USA;

**CSAC** California State Arthropod Collection, Sacramento, USA;

**EMEC**Essig Museum Entomology Collection, University of California, Berkeley, USA;

**NMNH**National Museum of Natural History, Washington, DC, USA;

**OSAC**Oregon State Arthropod Collection, Oregon State University, Corvallis, USA.

### ﻿Morphological methods

General methods of specimen preparation for morphological work, and terms used, follow Maddison (1993, 2008). Genitalia were prepared, after dissection from the body, by treatment in 10% KOH at 65 °C for 10 minutes followed by multi-hour baths of distilled water, 5% glacial acetic acid, distilled water, and finally 100% ethanol. Male genitalia were then mounted in Euparal between two small coverslips attached to archival-quality heavyweight watercolor paper, and, once dried, pinned beneath the specimen.

Photographs of entire beetles, as well as the head and pronotum pictures, were taken with a Leica M165C dissecting scope and a Sony NEX-7 camera, and of male genitalia with a Leica DM5500B compound microscope and DMC425C camera. Microsculpture photographs were taken with a DMC425C camera attached to a DM5500B compound scope equipped with an X-Cite 110LED light source, which provides co-axial illumination, and a 20× epi-illumination objective lens. For all photographs of specimens or body parts, a stack of images from different focal positions was merged using the PMax procedure in Zerene Systems’s Zerene Stacker; the final images thus potentially have some artefacts caused by the merging algorithm. Measurements were made using Leica Application Suite v. 4.9 from images acquired using either a Leica Z6 Apo lens and DMC4500 camera or a Leica DM5500B compound microscope and DMC425C camera.

### ﻿Taxon sampling for DNA studies

We obtained new DNA sequence data for the holotype of *Bembidionbrownorum* sp. nov. and 13 specimens of related species of *Bembidion*. These new data were combined with previously published data from six additional specimens (Maddison 2012; Sproul and Maddison 2017b). As *Bembidionflohri* Bates, *B.mormon*, *B.callens*, and *B.obtusangulum* appear to be the closest relatives of *B.brownorum* based upon a more extensive sampling of *Bembidion* (Maddison unpublished), we focused our sampling on those species, and included three additional species (*B.obtusidens* Fall, *B.scudderi* LeConte, and *B.consimile* Hayward) as outgroups (Tables 1, 2). All voucher specimens are deposited in OSAC except for the holotype of *B.brownorum* (deposited in EMEC) and the paralectotype of *B.callens* (specimen 4939, deposited in NMNH as specimen USNM.Ent.01114823).

**Table 1.** Sampling of members of Bembidion (Notaphus) for DNA-based study. Four-digit numbers under “#” are D.R. Maddison DNA voucher numbers, and an abbreviation for state or province of capture under “Loc”; further information on the newly sequenced specimens is given in Table 2. Other entries are GenBank accession numbers. Newly acquired sequences are those with accession numbers beginning with “OQ”.

	#	Loc	28S	COI	CAD	Topo
**Outgroups**						
*B.obtusidens*	2042		KY246703	KY246743	KY246784	KY246824
*B.scudderi*	1471		OQ286105	OQ284076	OQ288589	OQ288575
*B.consimile*	2506		OQ286106	OQ284077	OQ288590	OQ288576
***obtusangulum* group**						
*B.flohri*	3049	AB	KY246708	KY246749	KY246789	KY246830
*B.flohri*	3046	OR	KY246707	KY246748	KY246788	KY246829
*B.flohri*	1753	NV	JN170340	JN171035	JN170807	JN171216
*B.flohri*	3061	UT	KY246709	KY246750	KY246790	KY246831
*B.flohri*	5234	NM	OQ286107	OQ284078	OQ288591	OQ288577
*B.mormon*	3044	OR	OQ286110	OQ284081	OQ288594	OQ288580
*B.mormon*	2142	UT	OQ286111	OQ284082	OQ288595	OQ288581
*B.mormon*	3045	UT	OQ286112	OQ284083	OQ288596	OQ288582
*B.mormon*	4977	NV	OQ286109	OQ284080	OQ288593	OQ288579
*B.mormon*	2039	CA	OQ286108	OQ284079	OQ288592	OQ288578
*B.obtusangulum*	2051	AB	JN170397	MF616907	JN170869	MF616774
*B.obtusangulum*	3151	AB	OQ286113	OQ284084	OQ288597	OQ288583
*B.obtusangulum*	3594	CO	OQ286115	OQ284086	OQ288599	OQ288585
*B.obtusangulum*	3043	CA	OQ286114	OQ284085	OQ288598	OQ288584
*B.callens*	4936	AZ	OQ286116	OQ284087	OQ288600	OQ288586
*B.callens*	4939	AZ	OQ286117	OQ284088	OQ288601	OQ288587
*B.brownorum*	5864	CA	OQ286118	OQ284089	OQ288602	OQ288588

**Table 2.** Locality information for specimens of Bembidion (Notaphus) analyzed for DNA. Four-digit numbers under “#” are D.R. Maddison DNA voucher numbers.

Species	#	Locality
*B.obtusidens*	2042	Canada: Alberta: Burbank, junction of Red Deer and Blindman Rivers, 52.3542°N, 113.7556°W
*B.scudderi*	1471	Canada: Alberta: Bow River at highway 36, 50.246°N, 112.077°W
*B.consimile*	2506	USA: Colorado: Huerfano Co., Butte Road at I-25, 1825m, 37.7454°N, 104.832°W
*B.flohri*	3049	Canada: Alberta: Birch Lake, 640 m, 53.362°N, 111.5231°W
*B.flohri*	3046	USA: Oregon: Harney Co., Harney Lake, NE corner, 1237 m, 43.2750°N, 119.0902°W
*B.flohri*	1753	USA: Nevada: Lyon Co., Carson River near Weeks, 390 m, 39.2866°N, 119.2778°W
*B.flohri*	3061	USA: Utah: Salt Lake Co., Great Salt Lake Marina, 1280 m, 40.7482°N, 112.1856°W
*B.flohri*	5234	USA: New Mexico: Torrance Co., Laguna del Perro, 1861 m, 34.6003°N, 105.9252°W
*B.mormon*	3044	USA: Oregon: Harney Co., 00 Ranch Road NW Harney Lake, 1240 m, 43.2804°N, 119.1976°W
*B.mormon*	2142	USA: Utah: Salt Lake Co., Great Salt Lake Marina, 390 m, 40.7482°N, 112.1856°W
*B.mormon*	3045	USA: Utah: Salt Lake Co., Great Salt Lake Marina, 1280 m, 40.7482°N, 112.1856°W
*B.mormon*	4977	USA: Nevada: Mineral Co., Walker Lake, Twenty Mile Beach, 1200 m, 38.7503°N, 118.7577°W
*B.mormon*	2039	USA: California: Inyo Co., Owens Lake, 1100 m, 36.4684°N, 117.8585°W
*B.obtusangulum*	2051	Canada: Alberta: Kenilworth Lake, 10.vi.1993. DRM 93.054
*B.obtusangulum*	3151	CANADA: Alberta: High Level, 330 m, 58.5073°N, 117.1385°W
*B.obtusangulum*	3594	USA: Colorado: Alamosa Co., Alamosa NWR, 2292 m, 37.4435°N, 105.7722°W
*B.obtusangulum*	3043	USA: California: Mono Co., Mono Lake, 1940 m, 37.97780°N, 119.13000°W
*B.callens*	4936	USA: Arizona: Coconino Co., Havasu Indian Reservation, Havasu Springs, 36.2176°N, 112.6871°W
*B.callens*	4939	USA: Arizona: Tucson
*B.brownorum*	5864	USA: California: Colusa Co. Antelope Valley, Freshwater Creek, 39.13841°N, 122.34621°W

### ﻿DNA sequencing

Genes studied, and abbreviations used in this paper, are: **28S**: 28S ribosomal DNA (D1–D3 domains); **COI**: cytochrome c oxidase subunit I; CAD: part 4 of carbamoyl phosphate synthetase domain of the *rudimentary* gene; **Topo**: topoisomerase I.

For specimens collected into 95–100% ethanol (all but the paralectotype of *B.callens* DNA4939), DNA was extracted using a Qiagen DNeasy Blood and Tissue Kit. Fragments for the four genes were amplified using the Polymerase Chain Reaction on an Eppendorf Mastercycler Pro Thermal Cycler, using TaKaRa Ex Taq and the basic protocols recommended by the manufacturers. Primers and details of the cycling reactions used are given in Maddison (2012) and Maddison and Cooper (2014). The amplified products were then cleaned, quantified, and sequenced at the University of Arizona’s Genomic and Technology Core Facility using a 3730 XL Applied Biosystems automatic sequencer. Assembly of multiple chromatograms for each gene fragment and initial base calls were made with Phred (Green and Ewing 2002) and Phrap (Green 1999) as orchestrated by Mesquite’s Chromaseq package (Maddison and Maddison 2021a, c), with subsequent modifications by Chromaseq and manual inspection. Multiple peaks at a single position in multiple reads were coded using IUPAC ambiguity codes.

DNA extraction and sequencing of the paralectotype of *Bembidioncallens* DNA4939 followed Sproul and Maddison (2017b). In brief, DNA in that specimen was extracted using the Qiagen QIAmp Micro Kit (using the standard protocol with carrier RNA added), with dual-index libraries prepared using the NEBNext DNA Ultra II kit (New England BioLabs), which were then sequenced on an Illumina HiSeq 3000, multiplexed on a 150-base paired-end lane at the Oregon State University Center for Quantitative Life Sciences. No other members of subgenus Notaphus were included on that lane. Approximately 66 million reads were obtained for the sample. Reads were processed in CLC Genomics Workbench (CLCGW) v. 22.0. Reads were trimmed to eliminate low-quality ends (limit = 0.000316, corresponding to a quality score of 35) and to remove adapter sequences. The number of reads left after trimming was approximately 46 million. De novo assemblies were generated using CLCGW from paired, trimmed reads using an automatic word and bubble size, with the minimum contig length set to 200. The de novo assemblies were converted to BLASTable databases using NCBI’s makeblastdb tool and BLASTed using Mesquite’s (Maddison and Maddison 2021c) local BLAST tool (1E-30 as the e-value cutoff for nuclear protein-coding genes, and 1E-100 the cutoff for COI and 28S) using as query sequences the sequences of the four target genes from *B.mormon* DNA3045. For each gene, only one contig was returned; these hits were BLASTed to NCBI’s GenBank, and in all cases returned *Bembidion* as the top match.

Newly acquired sequences are all of “genseq-4” (Chakrabarty et al. 2013), except for those of the holotype of *B.brownorum* (specimen 5864) which are “genseq-1”, and those of the paralectotype of *B.callens* (specimen 4939), which are “genseq-2”.

### ﻿Alignment and data exclusion

COI, CAD, and Topo were easily aligned by eye, as there were no insertions or deletions (indels) evident in the sampled sequences. Alignment of 28S was conducted in MAFFT v. 7.130b (Katoh and Standley 2013) using the L-INS-i search option and otherwise default parameter values. In general, no sites were excluded from analyses, except at the 5’ and 3’ ends of the alignments; those regions were excluded, as they were mostly missing data, containing data from only a small fraction of the samples because of variation in the length of the sequences that were obtained.

### ﻿Phylogenetic analyses

A maximum-likelihood (ML) analysis was conducted for each gene individually using IQ-TREE v. 2.1.3 (Nguyen et al. 2015), as orchestrated by Mesquite’s Zephyr package (Maddison and Maddison 2021b, c). The ModelFinder feature within IQ-TREE (Kalyaanamoorthy et al. 2017) was used to find the optimal character evolution models. The MFP model option was used for 28S, and the TESTMERGE option for protein-coding genes. The TESTMERGE option sought the optimal partition of sites, beginning with the codon positions in different parts. Fifty searches were conducted for the ML tree for each matrix analyzed. In addition, analyses of a matrix formed by concatenation of all four gene fragments were conducted, with the TESTMERGE option also being used, beginning with each codon position for each gene as a separate part (thus, the analysis began allowing for up to 10 parts: three for each of the three protein-coding genes, and one for 28S). Fifty searches were conducted for the ML tree. For standard, non-parametric bootstrap analysis of the concatenated data, 500 replicates were used.

### ﻿Data availability

Sequences of the studied genes have been deposited in GenBank with accession numbers OQ284076 to OQ284089, OQ286105 to OQ286118, and OQ288575 to OQ288602. Files containing the untrimmed gene sequences for each specimen as well as the inferred trees for each gene have been deposited in Dryad (data available from the Dryad Digital Repository at https://doi.org/10.6078/D17416.

## ﻿Results

### ﻿Molecular and phylogenetic results

**Figure 2.**
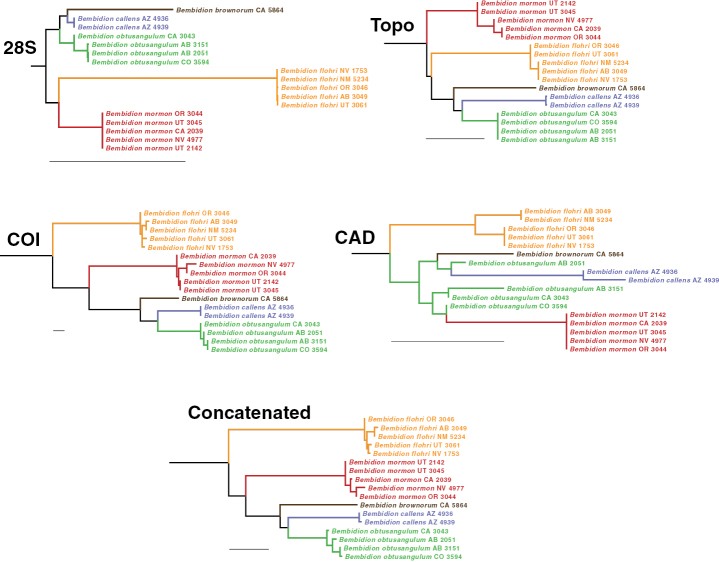
Maximum-likelihood trees of the *Bembidionobtusangulum* group for four individual genes as well as the concatenated matrix. Scale bars: 0.01 nucleotide substitutions per nucleotide site, as reconstructed by IQ-TREE. Outgroups not shown; in all single-gene and concatenated analyses, the five species shown here formed a clade, with a bootstrap value of 100% in the concatenated analysis.

The single specimen of *Bembidionbrownorum* sequenced is quite distinctive in DNA sequences of 28S, COI, and Topo, and clearly outside the bounds of sequence variation in the other species sampled. This is evident both by the gene trees (Fig. 2) and by the details of variation. *Bembidionbrownorum* differs from all specimens of the morphologically most similar species, *B.mormon*, at 13 bases in 28S, 44 bases in COI (6.7% divergence), two amino acids in CAD, and one amino acid in Topo. It differs from *B.callens*, which appears as a member of its sister group, at seven bases in 28S, 28 bases in COI (4.3% divergence), and three amino acids in CAD, and from the other member of its sister group, *B.obtusangulum*, at nine bases in 28S, and 33 bases in COI (5.0% divergence).

Three of the genes (28S, COI, and Topo) individually suggest that the nearest relatives of *B.brownorum* are *B.callens* and *B.obtusangulum*; in the ML tree of the concatenated matrix of all four genes, the latter two species form a clade with *B.brownorum* as its sister group (Fig. 2). The bootstrap value for the concatenated matrix for this sister-group relationship is 68%.

### ﻿Morphological results

**Figure 3.**
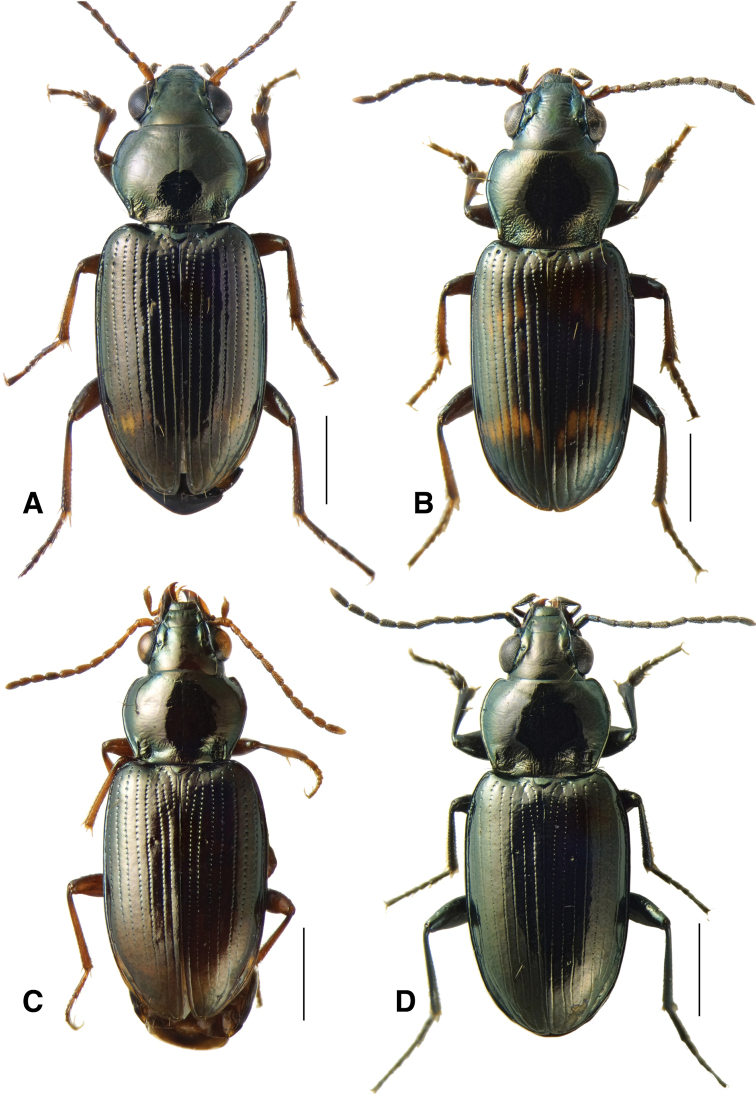
Habitus of members of the *Bembidionobtusangulum* species group **A***Bembidionbrownorum*, holotype male **B***Bembidionmormon* male **C***Bembidioncallens* (paralectotype) female **D***Bembidionobtusangulum* male. Scale bars: 1 mm.

*Bembidionbrownorum* has a distinctively convex and rounded prothorax (Fig. 3A) in addition to other characteristics that distinguish it from additional members of the subgenus Notaphus, as documented in the Taxonomic Treatment section below.

### ﻿Taxonomic treatment

To accommodate *B.brownorum*, couplet 169 in Lindroth’s (1963) key can be modified into a triplet as follows (figure numbers refer to those in Lindroth (1963) except as specified):

**Table:** 

169	3.5–4.0 mm. Upper surface unmetallic. Prothorax with evident latero-basal carina (fig. 171b)	**102. *B.nudipenne***
–	4.4–5.0 mm. Upper surface unmetallic or only slightly metallic. Latero-basal carina evident, long (this paper, Fig. 4B). Prothorax broad and very convex (this paper, Fig. 1). California	***B.brownorum***
–	4.3–5.9 mm. Metallic above. Latero-basal carina thin, usually rudimentary, or absent (fig. 187). Prothorax convex	**171**

#### ﻿ Bembidion brownorum

Taxon classificationAnimaliaColeopteraCarabidae

Maddison, Sproul & Will sp. nov.

75A896A5-49C7-555F-B6F4-B6CC5CF23903

https://zoobank.org/3B2A0EB4-D01A-4FBA-AE98-6898FDAF878D

##### Type materials.

***Holotype*.** Male, in EMEC, herein designated, labeled: “39.13841/−122.34621 USA: California: Colusa Co. Antelope Valley, Freshwater Creek uv light pan trap 133 m. 1.vii.2021 K.Will [Cal2021.vii.1.2]”, “David R. Maddison DNA5864 DNA Voucher” [pale green paper], “HOLOTYPE Bembidionbrownorum Maddison, Sproul, & Will” [partly handwritten, on red paper], “UC Berkeley EMEC 347587” [with matrix code on right side]. Genitalia mounted in Euparal in between coverslips pinned with specimen; extracted DNA stored separately. GenBank accession numbers for DNA sequences of the holotype are OQ284089, OQ286118, OQ288588, and OQ288602.

***Paratypes*.** (13 males, 8 females). “Borax Lake, Lower Lake, Lake Co., Cal. May 14 1922” (2, CAS). “Atwater, Merced Co., Calif 15 Aug 1966” (5, CAS, OSAC). “Wood L., Tulare Co., Calif. Rotary Trap V-22-1947 Norman W. Frazier, EMEC347588” (1, EMEC). “Wood L., Tulare Co., Calif. Rotary Trap V-24-1947 Norman W. Frazier EMEC347589” (1, EMEC). “Redondo, Cal.” (1, CAS). “Pasadena, Cal.” (3, CAS). “San Joaquin Mill Tulare Co., Calif. May 15, 3800 ft” (2, CAS). “Azusa, Cal.” (1, CAS). “Riverside, Cal. F.E. Winters” (1, CAS). “CALIF: Forest Home, San Bernardino Mts, 6000 ft. May” (1, CAS). “Poway, San Diego Co., Cal.” (2, CAS). “S. Cal” (1, CAS).

**Figure 4.**
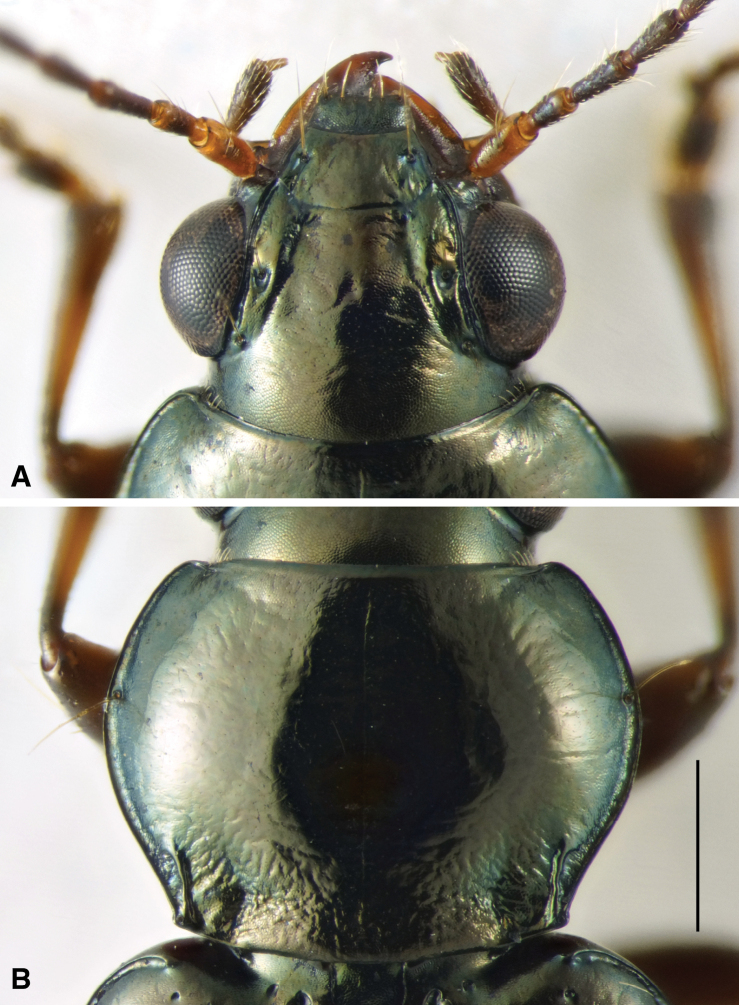
Head and pronotum of holotype of *Bembidionbrownorum*. Scale bar: 0.5 mm.

##### Type locality.

**Figure 5.**
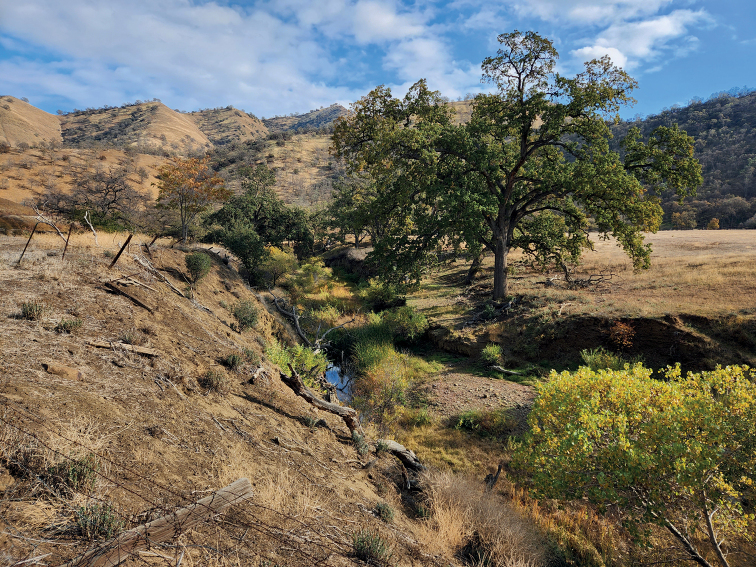
Type locality of *Bembidionbrownorum*. USA: California: Colusa Co. Antelope Valley, Freshwater Creek, 39.13841°N, 122.34621°W. Image taken November 2022.

USA: California: Colusa Co. Antelope Valley, Freshwater Creek, 39.13841°N, 122.34621°W (Fig. 5).

##### Derivation of specific epithet.

The specific epithet *brownorum* is treated as a noun in the genitive case and refers to Jerry and Anne Brown, former Governor and First Lady of California, respectively. The name is formed in their honor as it was their hospitality and openness to allowing access for research of insects on their ranch, the type locality, which led to the discovery of this species. Additionally, this honors their long commitment to environmentalism and continued efforts in the international climate-change movement.

##### Diagnosis.

A relatively large Bembidion (Notaphus), superficially similar to *B.mormon* (with which it has been confused in collections), with which it shares a pale subapical band on the elytra. However, *B.brownorum* has a much more convex pronotum, giving it an inflated appearance; the pronotum has more rounded sides and is more constricted posteriorly. From *B.callens* and *B.obtusangulum*, in addition to the prothorax shape, it is distinguished by presence of pale elytral spots, which those two species lack.

##### Description

**Figure 6.**
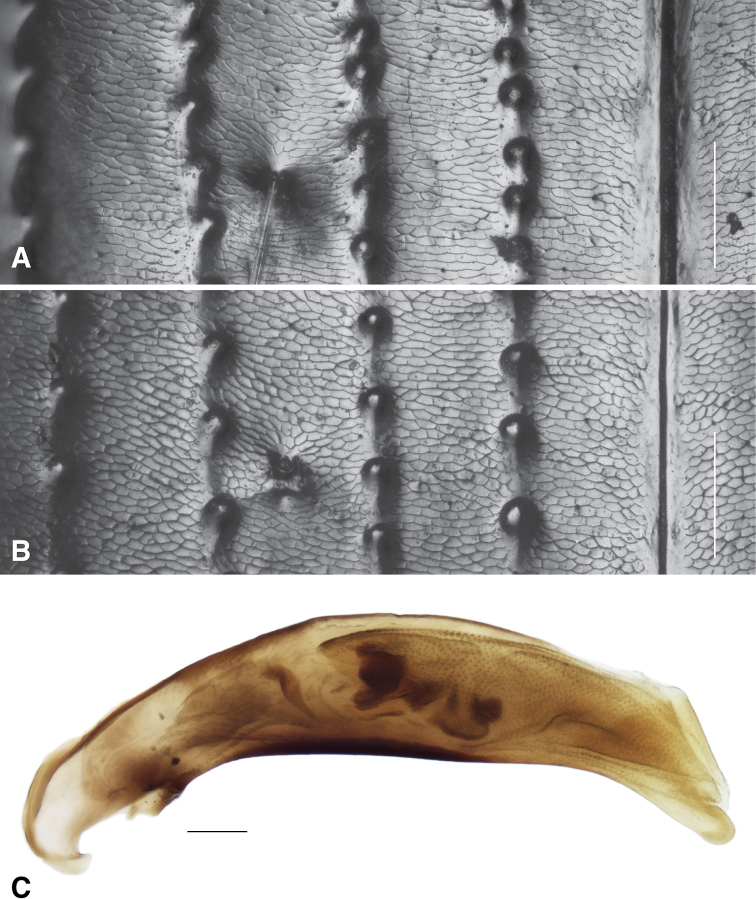
Microsculpture and aedeagus of *Bembidionbrownorum***A** elytral microsculpture around seta ed3 of holotype male **B** elytral microsculpture around seta ed3 of a female from Woodlake **C** aedeagus of holotype male. Scale bars: 100 µm.

**(based upon the holotype and 21 paratypes).** Body length 4.4–5.0 mm. Body dark brown or dark reddish brown, with head and pronotum slightly darker than elytra; elytra each with one diffuse pale spot at about the posterior fourth. Legs uniform in color, reddish brown; antennae brown, with first antennomere paler, at least ventrally. Mentum with anterior lateral regions large, with apical portion broadly rounded, not angulate; medial tooth simple (not bifid) with truncate tip; frontal furrows weakly defined, shallow; eyes prominent (Fig. 4A). Prothorax large, notably convex, with sides strikingly rounded such that the width at middle is much greater than the width at the posterior margin, with sides immediately front of hind angle slightly sinuate (Fig. 4B); hind angle slightly obtuse; posterolateral carina well defined, moderately long; posterior region of pronotum slightly rugose. Elytra with lateral bead not prolonged medially at shoulder; all striae complete, striatopunctate, with much smaller punctures in the posterior half. Microsculpture present on most of the dorsal surface of the body except for the disc of the pronotum, which is glossy; evident in both sexes over entire surface of elytra, consisting of sculpticells that are slightly transverse, more deeply engraved in females (Fig. 6B) than in males (Fig. 6A); female elytra thus matte. Pronotum with two lateral setae on each side; elytron with two setae in third interval. Aedeagus (Fig. 6C) typical for a member of subgenus Notaphus.

##### Flight ability.

All 18 specimens examined for wing condition are macropterous. The capture of two specimens from Woodlake in a rotary trap (Winkler 1949) and the capture of the holotype at a UV light both suggest that these beetles can fly.

##### Geographic variation.

None noted.

##### Geographic distribution.

**Figure 7.**
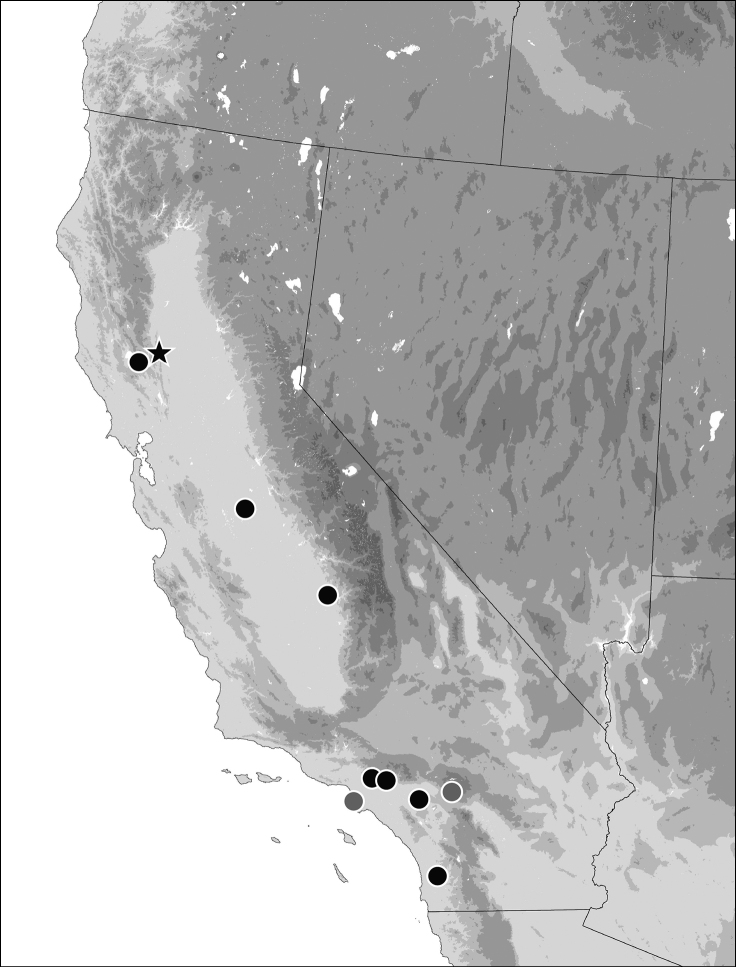
Geographic distribution of *Bembidionbrownorum* in California. The locality indicated with a star is the type locality. The two localities indicated by gray dots are uncertain or doubtful. Darker gray areas in the base map represent higher elevations.

Central Valley, Los Angeles Basin, and surrounding areas of California (Fig. 7). Two of the localities on the map are marked as uncertain: those labeled as from Forest Home and Redondo. The Forest Home locality is at much higher elevation than all other specimens (6000 ft). Based upon the labeling on other specimens from the F.E. Winters collection, the hand-written label attached to this specimen appears not to be an original label, and we have doubts about the validity of the data on the label. We have some doubts about the locality for the specimen labeled “Redondo”, as there are at least seven localities in California that include “Redondo” in the name (USGS Geographic Names Information System, https://edits.nationalmap.gov/apps/gaz-domestic/public/search/names). An additional locality, “San Joaquin Mill Tulare Co., Calif 3800 ft” was not mapped as we could not determine the site with any certainty.

##### Habitat.

The only specimen with detailed collecting data including an exact locality is the holotype. Because it was collected at a UV light, the specimen was not found in its natural microhabitat, and we do not know how far it had flown from a suitable habitat. However, the type locality might provide some hints about possible habitat of the species. The type locality lies on the east side of Antelope Valley in the northern part of the Cortina Ridge, which marks the western edge of the Colusa Basin region of the Sacramento Valley. The ridge is formed of tilted sandstone beds, mudstone, and siltstone formed from the eroded sediments of the Sierran–Klamath terrane. As members of subgenus Notaphus are almost universally found at the edges of bodies of water (with exceptions for some species found at high elevation), we expect *B.brownorum* to live on lake, pond, marsh, river, or creek shores. The UV light was set up next to Freshwater Creek (Fig. 5), which might be the habitat of the specimen. Freshwater Creek cuts through Cortina Ridge; its bed is composed of consolidated claystone and lenses of poorly hardened conglomerate, sandstone, and siltstone. The stream is somewhat trellis-like, with persistent pools, due to the presence of minor ridges of erosion-resistant materials. The current dominant vegetation consists of grasses, cattails, willows, and rushes near the stream. Sparsely set oaks line the stream edge and lateral drainages. Water is persistent and flows on the surface in portions of the stream throughout the year. Evaporation along the stream margin intermittently creates a hardened crust of white mineral deposits that often overlays a black, highly organic mud in depositional stretches. Narrow, steep-sided sections of the stream have banks composed of exfoliating claystone and mud from eroded topsoil. The adjacent land was historically used for crop production, e.g., barley, but the land and water has primarily been used for cattle ranching. Both the stream bed and adjacent area show the impact of many years of cattle grazing. There is an historical account of beaver damming (J. Brown pers. com.) but there is presently no impact of this event. There is no evidence that the water flow has been artificially dammed, channelized, or diverted in the collection area. As such, this stretch of Freshwater Creek represents a relatively unaltered, natural, perennial source of surface water—unusual in the Central Valley and adjacent foothills of California. However, we consider evidence for *B.brownorum* being a stream-shore species very weak, as it is based upon only one specimen which might have flown in from some distance.

The other known localities do not clearly suggest a specific habitat, nor do the known localities of related species. *Bembidionbrownorum* localities include a lake with sodium borate deposits (Borax Lake, Lake County), a city with a lake with extensive, flat shores (Bravo Lake in Woodlake), and a site near the Pacific Ocean that once had a salt lake (Redondo). At least the latter two are habitats similar to the saline, pond and lake shore habitats frequented by *B.obtusangulum* and *B.mormon*, near relatives of *B.brownorum*. The specimen labeled as from 6000 feet elevation at Forest Home, San Bernardino Mts (presumably around 34.083°N, 116.893°W) suggest instead a less saline, creek shore habit, but we doubt the veracity of that label (see above). The only specimen of the related *B.callens* with known habitat data is a specimen collected by Larry Stevens in gravel around the calcium-carbonate-rich waters of Havasu Springs, Arizona, at 36.2176°N, 112.6871°W (Larry Stevens pers. comm. 2022).

## ﻿Discussion

Much attention has recently been drawn to the apparent decline of insect populations (Sánchez-Bayo and Wyckhuys 2019; Wagner 2020; Wagner et al. 2021). Given the many, significant changes brought about by human activities, which range in scale from local and ephemeral to global and long-term, substantial changes in insect abundance and species assemblages are not surprising. Even in the absence of apparent changes in measures like species richness, insect assemblages may be homogenized with increases in generalist species and species that can take advantage of human habitat alterations (Ball-Damerow et al. 2014). Among carabid beetles, species decline and apparent extinction have been recently documented (Kotze and O’Hara 2003; Knisley and Fenster 2005; Brandmayr et al. 2009), and a number of traits have been hypothesized as being linked to decline and extinction risk (Kotze and O’Hara 2003; Nolte et al. 2019). Examples of these risk-linked traits are (1) habitat specialization, (2) small distributional range, (3) large body size, and (4) being either monomorphic macropterous or brachypterous. Bad luck (sensu Samways 2006) also plays a role, in that narrow habitat requirements or restricted ranges may coincide with human land use priorities that impact beetles. Among carabid beetles, there are notable examples of unlucky species from California. For example, the southern subspecies of the Golden Bear Harpaline (*Dicheirusdilatatusangulatus* Casey) is hypothesized to have been impacted by annual fire break disturbance (Noonan 1968), transformation of seasonal wetlands to agricultural production that reduced available habitat significantly impacted the Delta Green Ground Beetle (*Elaphrusviridis* Horn) (Arnold and Kavanaugh 2021), and urban development and general habitat degradation have ongoing effects on the Ohlone Tiger Beetle (*Cicindelaohlone* Freitag & Kavanaugh) (Knisley and Arnold 2013).

It is likely that *B.brownorum* is yet another unlucky carabid beetle species. After our initial collection and recognition of *B.brownorum* we searched relevant collections for additional specimens. The small number of specimens located were only found in the EMEC and CAS collections, both with significant quantities of older material, including substantial holdings from pre-1950 (https://essig.berkeley.edu/museum-history/, https://www.calacademy.org/scientists/entomology-information-page#history). Despite having considerable and important carabid beetle holdings, no specimens were found in the CSAC and BMEC collections, which almost exclusively have specimens collected after 1970. Prior to 2021, the most recent specimen is from Atwater, California, collected in 1966. The only other specimens with an explicit date are from the 1940s. The undated specimens have labels in styles that suggest they are at least as old or older than those with dates. For example, specimens of the F.E. Winters collection in CAS and EMEC are typically from collecting events in the early twentieth century, up to about 1930. Such a long gap in sampling may be indicative of species deterioration but can also be the result of a lack of sampling (i.e., “Wallacean extinction” of Ladle and Jepson 2008) or the lack of available taxonomic expertise (also a resource in significant decline). Various factors can affect repeat collection of a given species, such as range size, collecting effort, habitat access, and collectability (Ladle et al. 2011).

*Bembidionbrownorum*’s large range (Fig. 7) and small body size suggest that it should be resistant to decline, but its apparent monomorphic macroptery could be a risk factor (Kotze and O’Hara 2003; Scheffers et al. 2011; Nolte et al. 2019). Additionally, given its large range and that these beetles are attracted to lights, frequent collection would be expected even though its small size might cause it to be missed during visual-search collecting by general collectors and despite habitat access issues in California. Other species collected abundantly in light traps in 2021 from Colusa County, such as Stenolophus (Stenolophus) anceps LeConte, Stenolophus (Stenolophus) limbalis LeConte, and Pseudaptinus (Pseudaptinus) tenuicollis (LeConte), Bembidion (Trepanedoris) connivens (LeConte), Bembidion (Furcacampa) timidum (LeConte), and Bembidion (Notaphus) approximatum (LeConte), are represented in all the collections we surveyed, in very large numbers, and across the full distributional and temporal range. These observations suggest that *B.brownorum* has likely experienced a significant decline in population numbers, and that the lack of recently collected specimens is not simply because it has been missed or overlooked by collectors.

Though details on the microhabitat are unknown, it seems probable that habitat specialization and the degradation of that habitat led to this species’ decline. At a very general level, most of the locations at which *B.brownorum* was historically collected presently have no apparent, natural habitat, and are entirely highly developed (Atwater, Woodlake, Redondo [Beach], Pasadena, and Azusa) or are largely developed with little potential habitat (Riverside and Poway). Perhaps the Borax Lake region, which is only 33 km southwest of the type locality, holds the best odds for a persistent population. In addition, lakes near previous localities may also harbor populations (e.g., Bravo Lake, within 4 km of the Woodlake collection site, or East Park Reservoir, about 25 km NNE of the type locality, or Indian Valley Reservoir, about 17 km W of the type locality).

The recent collection of *B.brownorum* raises our hopes that the species persists in more locations, and we encourage efforts to sample for it, but we are concerned that the species still has the potential to disappear forever. Rediscovery or re-collection does not mean a species is doing well. As pointed out by Scheffers et al. (2011), “88% of rediscovered [vertebrate] species are currently threatened” and declining abundance leads to declining detectability, making the job of conservation even harder. Will populations of this, and other imperiled species, be located before the many sources of disturbance (e.g., invasive species, habitat loss, pesticide exposure, and climate change) drive them to extinction?

## Supplementary Material

XML Treatment for Bembidion brownorum
